# Short-term outcome after treatment of talocrural instability in cats using modified type II transarticular external skeletal fixation

**DOI:** 10.1038/s41598-024-57781-w

**Published:** 2024-04-02

**Authors:** Shaaban Gadallah, Mohamed El-Sunsafty, Ahmed Sharshar, Tarik Misk, Carolin Fischer, Rodja Jaehrig, Christian Feichtenschlager, Martin Kramer, Amal Hammad

**Affiliations:** 1 Department of Surgery, Anesthesiology and Radiology, Faculty of Veterinary Medicine, University of Sadat City, Sadat City, 32511 Egypt; 2 Department of Veterinary Clinical Sciences, Faculty of Veterinary Medicine, Jordan University of Science and Technology, Irbid, 22110 Jordan; 3 Department of Veterinary Clinical Sciences, Clinic for Small Animals Surgery and Radiology, Faculty of Veterinary Medicine, Justus-Liebig-University, Giessen, Germany

**Keywords:** Cat, Talocrural instability, Tarsal joint, Transarticular external skeletal fixator, Zoology, Medical research

## Abstract

Transarticular external skeletal fixation (TESF) is repeatedly used for temporary stabilisation of tarsal joint in cats. Hence, this study aimed to evaluate the use of temporary modified type II TESF for management of talocrural instability (TCI) in cats without joint arthrodesis and to rate short-term outcomes and complications. Medical records of all cats treated for TCI between January 2012 and December 2021 were reviewed. Information was collected including signalment, degree of lameness, type of TCI, accompanying soft tissue and bone injuries, and post-operative follow-up assessment including time of frame removal, complications, degree of lameness, range of joint motion and ankylosis. Surgical management didn’t involve debridement of the articular cartilage. Eighty-five percent of cats had satisfactory joint stability at the time of frame removal. Eighteen cats exhibited minor complications, six cats had major complications, and 8 cats showed persistent lameness. All cats showed reduction of joint motion range by 20°–30° directly after frame removal while returned to normal in 79% of cats 4 weeks later. Variable degrees of joint ankylosis were reported. In conclusion, this study supports the use of temporary modified type II TESF for management of TCI in cats without joint involvement as an excellent alternative to tarsal arthrodesis.

## Introduction

Feline tarsus has a complex anatomical structure. It is composed of talocrural, talocentral, calcaneoquartal, centrodistal and tarsometatarsal joints^1^. The motion of the tarsus is mostly dependent on the talocrural joint, which gains its stability from the medial and lateral malleolus along with the collateral ligaments that originate deeply under the malleolus^2^.

Lack of soft tissue support makes the tarsal joint highly vulnerable to various injuries, which range from mild lacerations and instability to complete luxation^3–6^. Luxation of the feline tarsus has been described in different forms including talocrural instability (TCI), calcaneoquartal, and tarsometatarsal luxation. TCI usually involves rupture of one or both of the collateral ligaments in combination with fracture of the medial or the lateral malleolus^6–9^.

The essential factors in repair of TCI are the reduction and restoration of the articular surface to its normal anatomical position while maintaining its normal range of motion, preventing its further damage, and achieving early joint mobilization (to limit the incidence of osteoarthritis)^10,11^. Several surgical techniques have been used for management of TCI in cats, such as internal repair in combination with external coaptation, transarticular external fixation (TESF) or plate arthrodesis^2,6,12–19^. The deep origin of the oblique branches of the collateral ligaments and the small sized tarsal bones in cats, makes primary ligament suturing, prosthetic ligament reconstruction, and bone plating are challenging^19–22^.

It is well-established that TESF is the preferred immobilization technique for temporary joint stabilization in cats^15,23,24^. It can be used alone or combined with different internal repair techniques^6^. TESF has been advocated for management of TCI when skin wounds, and shearing injuries are apparent. It allows easy access to the wound for regular dressing and preserves fracture site biology. Moreover, it is well tolerated by the animal, allows early weight-bearing, and provides satisfactory mechanical protection to the repaired joint^6,8,10^. Despite this, TESF still has its own complications including pin loosening and pin tract infection^9,22,24,25^.

For many years, tarsal arthrodesis has been used as the salvage procedure for treatment of TCI in small animals, particularly in cats^4,5,7–9,22,23^. The documented disadvantages of this technique are, loss of joint function, implant associated complications, and infection comorbidities with cancellous bone harvesting^2,13,15,21,22,24^. In our clinic, TCI in cats has been managed using modified type II TESF alone or combined with primary ligament suture, ligament prostheses, Kirschner wires (K-wire), or lag screw fixation without debridement of the articular cartilage. The purposes of this study were to describe modified type II TESF technique used in our clinic without induction of joint arthrodesis, in addition to rate the short-term outcome and complications accompanying its use.

## Materials and methods

This is a retrospective study that carried out as collaboration between departments of Surgery, Anesthesiology and Radiology, Faculty of Veterinary Medicine, University of Sadat City, Egypt and department of Veterinary Clinical Sciences, Clinic for Small Animals Surgery and Radiology, Faculty of Veterinary Medicine, Justus-Liebig-University-Giessen, Germany as part of enhancing cultural exchange between Egypt and Germany.

### Animals

The medical records of all cats that were referred to the clinic of small animal surgery at JLU-Giessen, Germany, from January 2012 to December 2021 and suffering from TCI and treated using modified type II TESF were reviewed. Data obtained from the medical records included signalment, causative agent, degree of lameness, and skin condition. Pre-operative ordinary and stress radiographs were performed to determine type and degree of TCI (lateral or medial instability or complete luxation), ligaments involvement, and accompanying injuries. Immediate postoperative radiographs were taken to ensure correct implant position, proper fracture reduction and joint stabilization^12,21^. Follow-up radiographs were taken at 4–12 weeks post-operatively. Ethical approval was waived by the local Ethics Committee of University of Sadat City, Egypt, in view of the retrospective nature of the study (study included data collection, sorting and analysis based on existent medical records) and all the procedures being performed were a part of the routine care.

### Anesthesia and surgical technique

A standardized anesthetic protocol was used. Cats were premedicated by intramuscular injection (I/M) of medetomidine (50 µg/kg) and morphine (0.2 mg/kg). Anesthesia was induced by intravenous injection (I/V) of ketamine (2.5 mg/kg) and diazepam (0.5 mg/kg) and maintained by isoflurane in 100% oxygen using a small animal circuit without re-inhalation of gases.

For the procedure, the anesthetized cats were placed in dorsal recumbency, and the area of the tarsal region was aseptically prepared. For all cats, modified type II TESF has been used as the primary tool for tarsal immobilization. According to the degree of joint luxation, and presence or absence of fracture, TESF has been used alone or combined with primary ligament suture, ligament prostheses, K-wire, or lag screw fixation (Table 1). The surgery didn’t involve debridement of the articular cartilage. In cases of open joints, swabs were taken from the wounds for microbial examination.

**Table 1:** Patient details, nature of tarsal injury, selected surgical technique and approaches.

Surgical technique (number of cases)	Mean (range) age/body weight (B.W)	Case description	Surgical approach
TESF alone (nine cats)	Age: 78 months (27–173) B.W: 4.7 kg (3.6–5.6)	Six cats had complete luxation and bilateral rupture of collateral ligaments Two cats had medial TCI with MCLR, One cat had lateral TCI with LCLR Skin perforation was present in 2 cats, there was no sign of skin perforation in 7 cats	Frame was fixed without joint exposure. Skin wounds were treated to heal by second intention
TESF + primary ligament suture (eight cats)	Age: 84 months (17–165) B.W: 4.4 kg (3.6–5.7)	Six cats had complete luxation with bilateral rupture of the collateral ligaments Two cats had lateral TCI with LCLR Skin perforation was reported in all cats	In case of complete luxation, the tarsal joints were exposed via lateral and medial skin incisions. While in cases of lateral instability the tarsal joints were exposed via lateral skin incision
TESF + primary ligament suture + lag screw (seven cats)	Age: 96 months (29–232) B.W: 5.6 kg (4.3–6.6)	Two cats had a complete luxation accompanied with MCLR and MMF) Five cats had a complete luxation accompanied with LMF and bilateral collateral ligament rupture Skin perforation was present in 4 cats, there was no sign of skin perforation in 3 cats	The tarsal joints were exposed via lateral and medial skin incisions
TESF + primary ligament suture + K-wire (three cats)	Age: 97 months (78–108) B.W: 5.9 kg (4.0–9.6)	All cats had a complete luxation accompanied with MCLR and LMF Skin perforation was present in 1 cat, there was no sign of skin perforation in 2 cats	The tarsal joints were exposed via lateral and medial skin incisions
TESF + K-wire (one cat)	Age: 47 months B.W: 3.8 kg (3.6–5.7)	The cat had a complete luxation with LMF and MMF, while there was no sign of skin perforation	The tarsal joint was exposed via lateral and medial skin incisions
TESF + lag screw + K-wire (one cat)	Age: 65 months B.W: 4.0 kg	The cat had a complete luxation with LMF and MMF, and the skin was perforated	The tarsal joint was exposed via lateral and medial skin incisions
TESF + ligament replacement (ligament adaptation + bone tunnel) (one cat)	Age: 168 months B.W: 3.8 kg	The cat had a medial TCI with MCLR while the skin was perforated	The tarsal joint was exposed via medial skin incision

*TESF* transarticular external skeletal fixation, *TCI* talocrural instability, *MCLR* medial collateral ligament rupture, *LCLR* lateral collateral ligament rupture, *K-wire* Kirschner wire, *MMF* medial malleolar fracture, *LMF* lateral malleolar fracture.

In cases where collateral ligament suture or prosthesis with or without internal fixation was planned, the surgical approach was accomplished via lateral or medial skin incision as required (Table 1). Ligament suturing was performed, when possible, in a locking loop pattern using PDS*.* Prosthesis of the collateral ligament was achieved via ligament adaptation in combination with malleolus bone tunnel using Daflon. Accurate joint reduction and alignment were assessed by stressed manipulations of the joint in mediolateral, dorsoplantar, and rotary planes followed by routine wound closure.

**Figure 1:**
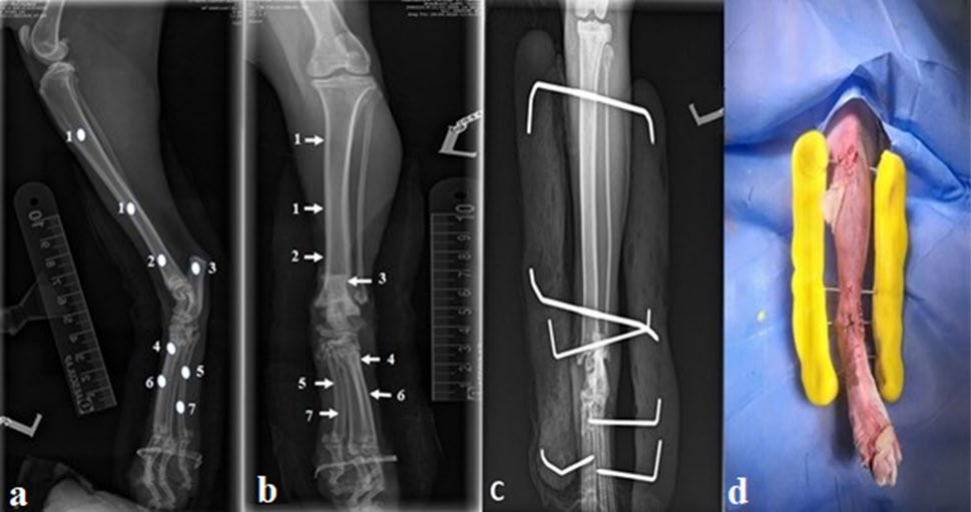
Mediolateral (**a**) and dorsoplantar (**b**) radiographs showing the fixation points of the frame. (**c**) Dorsoplantar post-operative radiograph of the frame after its fixation. (**d**) post-operative photograph showing the distance between skin and the connecting bars.

Frame fixation was also accomplished after repositioning the luxated joint in its normal anatomical position. According to the number of used pins, stab incisions were made on the skin at the medial aspect of the tibia (n = 2, the 1st at the proximal or the middle third while the 2nd at the distal third), lateral aspect of the calcaneal tuberosity (n = 1), and lateral and medial aspect of metatarsus (n = 3–4, two at the lateral and 1 or 2 at the medial aspect according to surgeon preference). The threaded part of the pins was engaged to the bone using a low-speed drill through pre-drilled bone holes. Center face positive profile threaded pins (1.6–2.5 mm) were fixed at tibial and calcaneus holes, while positive interface profile threaded pins (1.25–2.0 mm) were fixed at metatarsal bones whereas each pin passed through two metatarsal bones. The protruded ends of the pins were bent at a right angle, shortened, and joined to a bar of polymethyl methacrylate at 1–2 cm from skin surface while the tarsal joint was fixed at the range of the normal standing angle of the sound limb^1^ (Fig. 1). The space between skin and Technovit bar was packed with sterile dry cotton gauze and wrapped with protective bandage for 2–3 days to prevent postoperative swelling. Strict cage rest was recommended in all cases until frame removal (6–8 weeks)^25^. After which the tarsal joints were manually manipulated to assess the degree of joint stability and a crepe bandage was used for an additional two weeks to restrict joint movement.

### Post-operative assessment

Post-operative evaluation period was extended for four weeks after frame removal. The assessment included clinical evaluation, recording of post-operative complications, and the degree of joint ankylosis. Clinical evaluation included the degree of lameness (scored using a scale ranging from 0 to 4^26^), discharge at the site of pin entry, and the degree of joint mobility. The range of motion was evaluated directly after frame removal and 4 weeks later using the goniometric method^10^. Post-operative complications were defined as minor and major, as previously described^15^. The degree of joint ankylosis was assessed via radiographic evaluation of the final recheck radiographs in which the affected joint was compared with the contralateral limb. In all recheck radiographs, the degree of tarsocrural, intertarsal, and tarsometatarsal joint ankylosis was evaluated by two independent radiologists. They were asked to assess each joint alone and score it from 0 to 3. Where 0° means the joint is entirely normal. Degree 1 was defined as mild ankylosis with mild joint space narrowing without new bone formation. Degree 2 was defined as moderate joint ankylosis with moderate joint space narrowing with presence of small amount of new bone formation. While complete ankylosis with complete fusion of the tarsal bones was scored with degree 3.

### Statistical analysis

The obtained values were reported as mean and standard errors. A Kruskal–Wallis one-way analysis of variance with Dunn–Bonferroni post hoc tests were used for analyzing the obtained values and to determine the statistical relationship between animal’s signalment (age, weight and sex) and the used surgical technique in relation to post-operative lameness score, joint ankylosis, degree of joint motion. All values were analyzed using Grahpad prism software. For all tests, significance was set at P-value ≤ 0.05.

## Results

Thirty cats met the selection criteria and were included in the present study. The cats were European Short Hair (26), British Short Hair (1), Maine Coon (1), Persian Longhair (1), and one mixed breed. At the time of primary examination, the mean age of cats was 87 months (range 17–232 months) (Table 1). According to the range of age distribution, there were 6 young adults (12–36 months), sixteen mature adults (37–120 months), and eight geriatric (more than121 months)^11^. Twenty-seven cats were neutered (14 females and 13 male), and 3 were entire (one female and two males). The mean body weight was 4.8 kg (range 2.5–9.6 kg). Trauma was the main cause of TCI in 28 cats, while dog bite was reported to be the cause in 2 cats. Six cats presented with grade 3, and 24 cats with grade 4 lameness.

**Figure 2:**
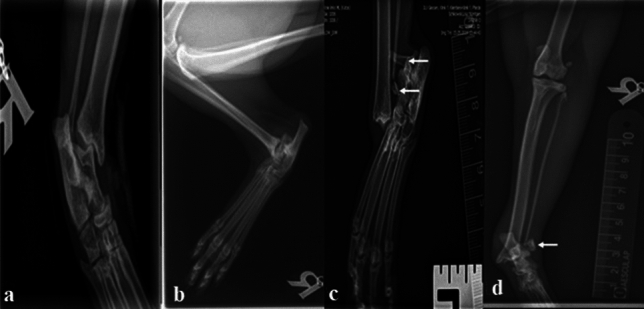
Dorsoplantar (**a**) and mediolateral (**b**) radiographs showing TCI. (**c**,**d**) showing complete luxation of the tarsal joint in two projections.

Unilateral TCI was observed in six cats (3 lateral and 3 medial) and complete luxation in 24 cats (Table 1 and Fig. 2). The skin was open in 17 cats, and intact in 13 cats. Malleolar fracture has been recorded in 12 cats; ten had lateral, and two had concomitant medial and lateral malleolar fractures (Fig. 2). Collateral ligament rupture was reported in 28 cats (fourteen medial, three lateral, eleven bilateral) (Table 1). Accompanying orthopedic injuries were reported in four cats. It included jaw fracture (n = 1), metatarsal fracture of the contralateral limb (n = 1), sacroiliac dislocation, iliac fracture, femoral neck fracture (n = 1), and hip luxation (n = 1). Bacteriological culturing was positive in three cases. *Pasteurella multocida* has been isolated from one cat*, Pseudomonas aeruginosa* has been isolated from the second while*, Aerobic bacilli, Enterococcus mundtii, and Pasteurella multocida* have been isolated from the last one.

**Figure 3:**
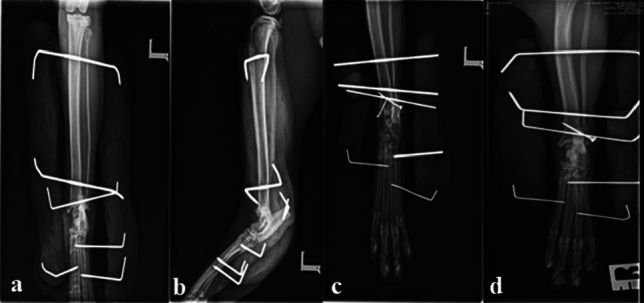
Radiographs showing the use of type II TESF alone (**a**,**b**) or in combination with a K-wire (**c**) or a lag screw (**d**).

TESF was used alone to manage TCI in nine cats without malleolar fracture. In the rest of the cases (n = 21), TESF was used in combination with a lag screw or K-wire with prosthetic ligament suture. Primary ligaments suture was performed in 18 cats. K-wire was used in 5 cats while, lag screw was used in 8 cats (Fig. 3). Ligament replacement was used in 1 cat (Table 1).

### Post-operative follow-up

**Figure 4:**
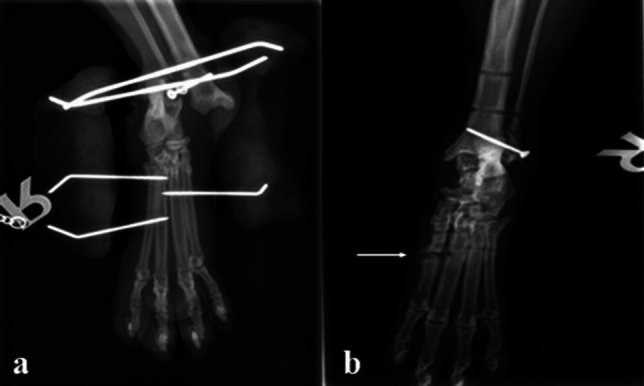
Radiographs showing post-operative complications after fixation of type II TESF including re-luxation of the talocrural joint (**a**) and fracture of the second metatarsal bone (**b**) (arrow).

The post-operative follow-up data of 4 cats were missed due to lack of owner compliance and failure to return for follow-up. Complications have been recorded in 24 (92%) of 26 cats. Eighteen cats (69%) exhibited minor complications including soft tissue swelling, skin necrosis at calcaneal tuberosity, muscular and bone atrophy, pin loosening, and pin tract infection. Such complications have been resolved with the administration of antibiotics and anti-inflammatories. Six (23%) cats exhibited major complications: including re-luxation (n = 4). Three of them were re-stabilized with TESF in combination with bone tunnel and suture fixation. The affected limb of the fourth cat was amputated. Another limb was amputated five days post-operatively due to infection. Fracture of the second metatarsal bone was detected in one cat 8 weeks post-surgery while fracture healing was in the remodeling phase (Fig. 4). After frame removal, 21 of 24 cats were considered to have satisfactory joint stability. In the remaining three cats (which suffered joint re-luxation), the tarsal joints were unstable.

The degree of lameness, tarsal ankylosis and joint movement angles were evaluated in 24 cats. By the end of the observation period, lameness score showed no significant differences in relation to animal’s age, weight, and sex (Table 2). At the time of frame removal, eight cats showed mild to moderate degree of lameness (3 cats had grade 1 and 5 had a grade 2 lameness) which persisted till the end of the observation period. The remaining cats (n = 16) could bear weight on their affected limbs without apparent lameness. The degree of tarsal movement was reduced by 20°–30° in all cats at the time of frame removal. At the end of the observation period, tarsal movement significantly improved and returned to its normal range in 19 cats, while it remained reduced in five cats.

**Table 2:** Effect of physiological factors (age, weight and sex) and surgical treatments on post-operative lameness score in injured cats.

Parameters	Classification	Post-treatment lameness score
Age	Less than 5 years	0.44 ± 0.17
	More than 5 years	0.94 ± 0.26
	P-value	0.13
Weight	Less than 4 kg BW	0.75 ± 0.17
	More than 4 kg BW	0.55 ± 0.18
	P-value	0.16
Sex	Male	0.61 ± 0.21
	Female	0.69 ± 0.17
	P-value	0.39
Surgical treatment	Treatment I (TESF alone)	0.85 ± 0.26
	Treatment II (TESF with internal repair)	0.73 ± 0.23
	P-value	0.22

Values are presented as mean ± SE.

**Figure 5:**
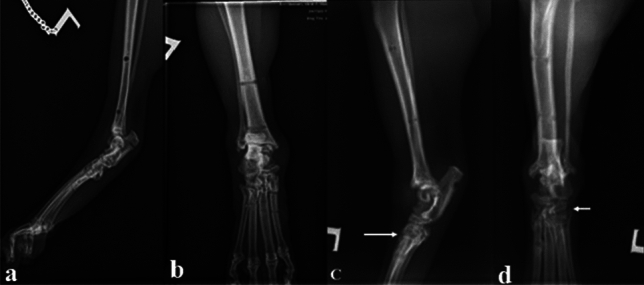
Radiographic view of the tarsal joints post-frame removal. In (**a**,**b**), the tarsal joints appeared normal without ankylosis. In (**c**,**d**), showing complete ankylosis of the tarsometatarsal joint (arrow).

Regarding the degree of tarsal ankylosis, there was only a significant relationship between the degree of ankylosis at talocrural joint and animal’s age. Older cats over 5 years of age showed higher degree of ankylosis compared to younger ones (Table 3). The talocrural joint of 8 cats appeared normal (score 0) in the radiographic examination, fifteen cats had a mild degree (score 1) of ankylosis, and one cat had a moderate degree (score 2) of ankylosis. The intertarsal joint appeared normal (score 0) in 10 cats, fourteen cats had mild degree (score 1) of ankylosis. The tarsometatarsal joint appeared normal (score 0) in 9 cats, nine cats had a mild degree (score 1) of ankylosis, and five cats had a moderate (score 2) degree of ankylosis, and one cat had severe degree of ankylosis (score 3) (Fig. 5).

**Table 3:** Effect of physiological factors (age, weight and sex) and surgical treatments on post-operative joint ankylosis score in injured cats.

Parameters	Classification		Post-treatment joint ankylosis score				
			Tarsocrural		Intertarsal		Tarsometatarsal
Age	Less than 5 years		0.38 ± 0.20^b^		0.38 ± 0.20		0.55 ± 0.29
	More than 5 years		0.79 ± 0.11^a^		0.73 ± 0.13		0.71 ± 0.12
	P-value		0.04		0.07		0.15
Weight	Less than 4 kg BW		0.79 ± 0.15		0.70 ± 0.19		0.83 ± 0.28
	More than 4 kg BW		0.47 ± 0.12		0.53 ± 0.13		0.67 ± 0.16
	P-value		0.06		0.22		0.45
Sex	Male		0.53 ± 0.16		0.53 ± 0.15		0.73 ± 0.18
	Female		0.76 ± 0.13		0.69 ± 0.17		0.76 ± 0.25
	P-value		0.14		0.25		0.42
Surgical treatment	Treatment I (TESF alone)		0.50 ± 0.18		0.64 ± 0.23		0.50 ± 0.18
	Treatment II (TESF with internal repair)		0.71 ± 0.12		0.60 ± 0.13		0.84 ± 0.19
	P-value		0.38		0.46		0.43

Values are presented as mean ± SE. Values within the same column with different superscripts are statistically (p ≤ 0.05) different.

## Discussion

Several reports have described TESF as the sole fixation method for management of TCI in small animals, especially in cats^9,15,24,25^. In the study reported here, preservation of the articular cartilage and management of TCI in cats using modified type II TESF alone or in-combination with other means of internal repair have provided satisfactory results. This management method reduced the overall complication rates and the degree of tarsal ankylosis. After frame removal, most of the treated cats restored their full limb function within a short period of time relative to previous studies^7,9,15^.

In this study, data regarding animal signalment was comparable to those reported in previous studies^25,27^. In a previous report by Kulendra et al.^15^, the authors have reported a higher proportion of males being involved in road traffic accidents comparing to females. In contrast, in our study, males and females are presented in equal numbers, with young and mature adults being more commonly affected than other age groups. These results were compatible with previous reports by Bradshaw^28^ and Barrat^29^ in which the authors reported that the home range of cats is closely related to animal’s age rather than its gender. They also noted that male and female cats nearly have the same home range size.

Tarsal injuries in cats are variable and frequently complex. In most cases, the degree of joint damage can be only evaluated intraoperatively^30^. In the present study, approximately 80% (24/30) of the treated cats, suffered complete luxation of the talocrural joint accompanied with ligament rupture (93.3%), open skin wounds (63.3%), and malleolar fracture (40%). These findings could reflect the complex anatomical nature of the tarsal joint upon which determination of the appropriate stabilisation technique and the concomitant complications are dependent^2,6,20–22,25,31^.

Partial or complete arthrodesis of the tarsal joint has been previously addressed^9,20^. Although pantarsal arthrodesis is often considered the salvage option for management of TCI in many circumstances, its application is usually accompanied by several technical difficulties and complications^4,5,20,22,31–33^. In this study, we used external supplementary support in the form of modified type II TESF for temporary stabilization of the talocrural joint in all cats on its own or to protect surgical stabilisation techniques such as prosthetic ligament suture. Surgical management of the luxated joints did not involve debridement of articular cartilage. Our goals were to minimally disrupt the traumatized tarsal joint, reduce the possibility of joint ankylosis and maintain normal joint movement to optimize outcome^24,31–34^.

The pins are inserted through predrilled holes to reduce complications associated with pin insertion^34^. The number of pins inserted at the tibia, calcaneal tuberosity and metatarsal bone was 2 + 1 + 3(4), respectively. In previous studies by Kulendra et al.^15^, Hammer et al.^25^ and Moon et al.^9^, center face positive profile threaded pins were inserted in metatarsal bones. It’s technically challenging to use all metatarsal bones as a point of fixation at the same time due to its anatomical characterization (the metatarsal bones being arranged in a dome shape). To overcome this difficulty, we used 3 or 4 interface positive profile threaded pins at the proximal and middle of metatarsal bones, where each pin passed through two metatarsal bones. Insertion of pins in such way allowed the use of all metatarsal bones as point of fixation which enhanced joint stability. The number of pins that were inserted across the tibia was reduced to 2 pins as recommended in previous studies^1,25^. In contrast, in another study by Kulendra et al.^15^, it has been recommended to use a minimum of three pins proximal and distal to the talocrural joint to reduce the incidence of implant-related complications. In our study, no implant failure in any of the treated case was reported. We found that use of two pins proximal to the hock joint was satisfactory, didn’t interfere with ambulation, and provided sufficient stability. In this study, to maximize talocrural stabilisation, calcaneal tuberosity was used as an additional fixation point. To our knowledge, this may be the first report to mention this point in cats. In only one canine case report by Mclennan^13^, calcaneal tuber was used as a fixation point during stabilisation of tarsometatarsal luxation. This was attempted to prevent rotation of the talus relative to the calcaneus which subsequently eliminated the need for the use of an additional pin through the body of the talus.

Previous studies by Owen^12^, Kulendra et al.^15^ and Hammer et al.^25^, used traditional clamp-and-rod system in which the connecting bar was made of stainless steel. These studies reported the drawbacks of the used system in terms of difficulty in its modulation and post-operative mediolateral radiographic assessment, its bulkiness, and weight which was cumbersome for cats. To overcome these issues, in the present study, polymethyl methacrylate was used as a connecting bar as it is light, easily moldable, radiolucent allowing postoperative radiographic evaluation, and facilitating the use of various sized pins^35^.

In canine shear injuries TESF has been used as a sole treatment to provide sufficient joint stabilisation until complete healing of the periarticular tissues with fibrous tissue formation^9,14,24^. In this study, TESF has been used as a sole-fixation device in 9 cats without concomitant malleolar fracture (seven had intact skin and 2 had open skin wounds). Our aims to use an external fixator alone in closed cases are to prevent articular cartilage damage and reduce the overall complications rate (especially those which are not fixator-associated). In other cases, in which the skin was open, the collateral ligaments were subjected to shear injures, and it was not possible to place ligament sutures or prostheses due to the small size of the bone fragments. With exclusion of frame-associated complications, the overall clinical results in such cases were satisfactory and similar to those in which the ligament was repaired in combination with temporary immobilization^24^**.**

It has been recommended that primary ligament repair should be accomplished as the treatment of choice whenever possible. Otherwise, prosthetic ligament reconstruction may mimic the ligament's action and ensure joint stability while allowing a suitable range of motion^2,10,18,36^. In this study, TESF was combined with primary ligament repair in 18 cats and with ligament prostheses in one cat. Whereas multifilament materials were reported to induce high rate of postoperative infection when used for ligament replacement^37^, we used absorbable and non-absorbable monofilament suture materials for ligament suture and prostheses, respectively as recommended in previous studies by Beever et al.^24^ and Hammer et al.^25^. Although in this study the long-term outcome after ligament suture was not assessed, neither complications directly attributable to ligament repair nor the used suture material have been reported.

Schmokel and Ehrismann^38^ reported that a 3–6-week period of rigid fixation of the tarsal joint was sufficient to form fibrous tissue adequately strong to fully support the joint. In the present study, TESF frame was applied for 6–8 weeks which was adequate for healing of malleolar fracture and achieving complete joint stabilisation in 81% (21/26) of cats. In this regard, the results of this study support previous reports by Earley and Dee^30^, Voss et al.^33^, Jaeger et al.^10^ and Hammer et al.^25^, in which the authors reported that long-term, rigid fixation should be avoided because of its harmful effect on joint physiology which in turn leads to articular cartilage degradation.

Following tarsal stabilisation, persistent lameness and reduction of joint mobility are constant clinical findings during the post-operative follow-up period in dogs and cats^10,24,25,32,38^. Although tarsal mobility range decreased by 20°–30° in all cats at the time of frame removal, it didn’t affect the movement patterns in most of them. By the end of the observation period, the percentage of lame cats reduced to 20.8% (5/24), while 79% (19/24) of cats showed normal range of motion in their joints. Schmokel and Ehrismann^38^, Jaeger et al.^10^ and Hammer et al.^25^ have attributed the reduction of joint mobility range to the extent of the initial joint injury, post-traumatic osteoarthritis, and the formed periarticular fibrosis. They have recommended early joint mobilization and physiotherapy to improve the quality and orientation of the newly formed collagen fibers, accommodate joint's biomechanical forces, and improve postoperative outcomes.

Although the articular cartilage was preserved in all animals, a variable degree of tarsal ankylosis was observed. However, it didn’t result in complete osseous union in the joints of all treated cats. Moreover, it didn’t markedly alter joint movement and the weight-bearing capacity of the affected limb^13^. The degree of ankylosis reported in this study might be attributed to joint immobilization and periarticular fibrosis resulting in fusion of low-motion tarsal joints with subsequent intraarticular adhesions and atrophy of articular cartilage^39,40^. The radiographic signs of ankylosis included joint space narrowing and new bone production as previously described by Brunnberg^39^. Although the evaluation criteria were discretionary and may differ from one to another, this may be the first study of its kind. The obtained results facilitated evaluation of the fixation technique and assessment of different radiographic changes between joints in all treated animals.

The overall complication rate reported in this study was 92%. A high proportion (69%) of which was defined as minor complications, while major complications were reported in 23% of cats. This is in accordance with previous reports in which the complication rate was up to 92%^10,12,15,22–25^. Most of the reported minor complications were fixator-associated. Such complications were manageable and self-limiting after frame removal^9,24^. Following exclusion of fixator-associated complications, the most severe reported complication was limb amputation (2 cats) due to re-luxation and infection.

The fundamental limitations of this study were its retrospective nature, loss of follow-up data of 4 cases due to the referral character of our clinic and post-operative management of most of the treated cats primarily by the referring veterinarians rather than by our clinic. Absence of long-term follow-up evaluation and objective gait assessment were another limitations.

In conclusion, usage of TESF along with preservation of the articular cartilage reduced the overall rate of tarsal ankylosis and preserved joint function. The used modified type II TESF frame was well-tolerated by the treated cats and facilitated post-operative follow-up. Some immediate post-operative complications due to the external fixator were reported. Despite of this, these complications were of minor severity and completely resolved. Considering this as well as the overall high success rate, we still suggest using type II TESF for stabilisation of tarsal joint in cats. This technique could be a better alternative to tarsal arthrodesis for management of TCI or luxation in cats.

## Data Availability

All data are included in this published article.
